# Native Bacteria Associated with Mushroom Cultivation Promote Mushroom Growth Through Multiple Mechanisms

**DOI:** 10.3390/microorganisms14061181

**Published:** 2026-05-24

**Authors:** Ke Li, Huifang Zhao, Di Meng, Xinlei Zhang, Zemin Fang, Juanjuan Liu

**Affiliations:** 1 School of Life Sciences and Medical Engineering, Anhui University, Hefei 230601, China; 17856680753@163.com (K.L.); zhf111398@163.com (H.Z.); dimeng2022@163.com (D.M.); xinlei_zhang24@163.com (X.Z.); 2 Anhui Key Laboratory of Biocatalysis and Modern Biomanufacturing, Hefei 230601, China; 3 Anhui Provincial Engineering Technology Research Center of Microorganisms and Biocatalysis, Hefei 230601, China

**Keywords:** *Pleurotus ostreatus*, bacterial isolates, mycelial growth promotion, dual culture, volatile organic compounds, *Ganoderma lucidum*, *Pleurotus pulmonarius*

## Abstract

Some microorganisms present in the cultivation environment serve as biocontrol agents and contribute to enhanced mushroom production. However, the native bacteria naturally associated with commercial cultivation bags of *Pleurotus ostreatus*, as well as their growth-promoting roles and underlying mechanisms, remain poorly understood. This study aimed to identify native bacteria that promote *Pleurotus ostreatus* development and to investigate the underlying mechanisms. Four native bacteria, including *Brevibacterium epidermidis* (P6), *Acinetobacter soli* (A7), *Pseudomonas parafulva* (A8), and *Pseudomonas hunanensis* (A12), were isolated based on their ability to promote mycelial growth of *P. ostreatus. B. epidermidis* P6 shortened complete mycelial colonization time from ~30 d to 14 d in dual cultivation bags. All four strains increased fresh mushroom yield, with *B. epidermidis* P6, *A. soli* A7, and *P. parafulva* A8 increasing the number of basidiomata, while *P. hunanensis* A12 enhanced their size. These strains produced exopolysaccharides that enhanced mycelial growth. Additionally, *B. epidermidis* P6, *A. soli* A7, and *P. parafulva* A8 also secreted extracellular crude proteins that also promoted mycelial growth. Bi-plates and further gas chromatography–mass spectrometry analysis demonstrated that volatile organic compounds from *P. hunanensis* A12, including acetone, 2-butanone, benzaldehyde, and 1-undecene, enhanced fungal mycelial growth. The mycelial growth rates of *Ganoderma lucidum* and *Pleurotus pulmonarius* were also enhanced by these four strains. These results reveal that four native bacterial strains promote mushroom development through complex mechanisms.

## 1. Introduction

Mushroom cultivation refers to the controlled growth and basidioma of various fungal species in soil or nutrient-rich substrates that support their development [1]. During their growth cycle, environmental factors such as temperature, humidity, and substrate pH significantly influence mycelial development. Furthermore, both wild and cultivated mushrooms interact with various microorganisms throughout their life cycle [2]. These interactions often involve direct physical contact and molecular exchanges mediated by signaling molecules, which play a crucial role in the exchange and transformation of metabolites between organisms and their surrounding environment [3]. In commercial mushroom cultivation, growth substrates are typically subjected to pasteurization. Many microbial strains that survive high-temperature treatment can become dominant and may exert growth-stimulating effects. These microorganisms transform the raw substrate into a more selective and nutrient-rich medium that is conducive to mushroom development. Furthermore, these microbial communities not only enhance mycelial growth but also facilitate primordium formation and improve substrate colonization [4].

The application of growth-stimulating microorganisms to enhance crop growth and yield through biological approaches has been widely employed in ecological and agricultural cultivation worldwide. Beneficial microorganisms not only promote crop growth rates but also contribute to the maintenance of crop health and development [5]. Several microbial populations have been identified as playing a crucial role in various stages of mushroom cultivation, particularly during mycelial growth, with most studies focusing on the genus *Agaricus* [1]. For example, *Pseudomonas* spp. have been shown to positively influence mycelial growth and basidiospore development [6]. The incorporation of biomycorrhizal additives into cultivation media has also been found to enhance the growth rate of *Agaricus* mushrooms [7]. In recent years, studies have increasingly focused on other fungal genera such as *Pleurotus* and *Tricholoma* [4,8,9,10]. The interactions and nutrient dynamics between mushrooms and their associated growth-promoting bacteria have been well documented. These interactions are leveraged to meet fungal growth requirements by transforming bacterial metabolites, stimulating extracellular enzymes for substrate degradation, and suppressing pathogenic microorganisms [3,10,11].

In addition to direct contact and metabolite exchange, volatile organic compounds (VOCs) produced by bacteria can also influence the growth of fungi. VOCs are low-molecular-weight substances characterized by high vapor pressure and low boiling points, which enable them to diffuse through soil and air over considerable distances. These physicochemical properties make VOCs particularly suitable as signaling molecules that can affect the growth and development of other organisms [12]. The bacteriostatic properties of VOCs are commonly observed [13,14], whereas their growth-promoting characteristics on mushrooms have been reported only recently. Briard et al. demonstrated that VOCs emitted by *Pseudomonas aeruginosa* significantly enhanced the biomass of the fungus *Aspergillus fumigatus*, with dimethyl sulfide identified as the primary compound responsible for this growth stimulation [15]. Similarly, Axel Orban et al. revealed that VOCs released by *Bacillus sphaericus* M48F promoted mycelial growth of *Pleurotus ostreatus* [10]. However, the growth-promoting effect was found to be species-specific, and 2,5-diisopropylpyrazine was suggested as one of the substances involved in this process.

*P. ostreatus* is one of the most widely cultivated edible mushrooms in the world [16]. It is a rapidly growing white-rot fungus capable of thriving on substrates with varying C/N ratios, including sawdust, rice straw, sugarcane bagasse, corn stover, waste cotton, wheat straw, and banana leaves [17]. Despite the known growth-promoting bacteria from other systems, the identity and functional mechanisms of growth-promoting bacteria native to the *P. ostreatus* substrate have not been systematically characterized. In this study, we isolated native bacterial strains from commercial cultivation bags colonized by *P. ostreatus* mycelia. We hypothesized that these native bacteria promote mycelial growth through multiple mechanisms, including the secretion of extracellular active substances (proteins and exopolysaccharides) and the release of specific VOCs. To test this hypothesis, we screened those that enhanced the mycelial growth of *P. ostreatus* through co-cultivation. Additionally, mycelial package experiments were conducted to evaluate whether these bacteria could shorten mycelial growth time and improve substrate utilization efficiency. Extracellular crude proteins and exopolysaccharides (EPSs) were extracted from the promoting bacteria to preliminarily investigate the potential mechanisms underlying mycelial growth enhancement. To evaluate the impact of bacterial VOCs on fungal growth, the bi-plate systems were combined with gas chromatography–mass spectrometry (GC-MS) analysis. Finally, to explore the potential broad-spectrum activity of the selected bacterial strains, we also tested their mycelial growth-promoting effects on *Ganoderma lucidum* and *Pleurotus pulmonarius* strain Jinxiu.

## 2. Materials and Methods

### 2.1. Bacterial Isolation, Selection and Identification

Bacterial isolation substrate samples were collected from *P. ostreatus* cultivation bags at the half-colonization stage in Fuyang Mushroom Factory, Anhui Province, China. The half-colonization stage was defined as the stage at which the mycelium had colonized approximately 50% of the substrate volume, as judged by visual inspection through the bags. Sample collection: three bags were sampled; samples were taken from the colonized zone (5 cm behind the growing front) using sterile forceps. Approximately 10 g of sample was placed in 100 mL of sterile water under aseptic conditions and subjected to sonication for 3 min using a high-intensity ultrasonic liquid processor to isolate bacteria that were tightly adhered to the mycelium and substrate surface. Subsequently, the samples were incubated on a shaker at 37 °C for 2 h. The supernatant was then aspirated, diluted, and spread onto Luria-Bertani (LB; 1 L, 10 g tryptone, 5 g yeast extract, 10 g NaCl, 10 g agar powder) medium. The inoculated plates were incubated with plates inverted in an incubator at 37 °C for 24 h. Following this, individual colonies were selected and subjected to repeated streaking for isolation and purification until the morphological characteristics of the bacterial isolates were completely consistent under a Zeiss microscope (Carl Zeiss Microscopy GmbH, Jena, Germany) with 1000× magnification. Bacterial strains were stored in glycerol stocks at −20 °C.

Selection of isolates for further screening. Ten out of 24 obtained isolates were selected based on preliminary co-culture tests with *P. ostreatus* (see Section 2.2). The isolates selected for further screening were those showing no obvious pathogenic potential and the ability to promote the mycelial growth of *P. ostreatus*. The other 14 isolates were discarded.

Identification of Bacterial Strains. To identify isolated bacterial species, single colonies of each bacterium were selected, and genomic DNA was extracted using the Bacterial Genome Extraction Kit (Beyotime Biotech, Shanghai, China) according to the manufacturer’s instructions. Subsequently, the extracted DNA was used as a template for PCR amplification of bacterial colonies. The PCR reaction mixture consisted of 1.0 μL of 27F and 1492R primers (Sangon Biotech, Shanghai, China) [18], 1.0 μL of diluted bacterial strains, and 22.0 μL of distilled water. Thermal cycling conditions included an initial denaturation step at 95 °C for 10 min, followed by 35 cycles of denaturation at 95 °C for 40 s, annealing at 55 °C for 40 s, and extension at 72 °C for 60 s. The full-length 16S rRNA gene was amplified using the universal bacterial primers 27F and 1492R. The resulting PCR products were sent to General Biological (Chuzhou, Anhui, China) for sequencing. The obtained sequence data were analyzed using EZBioCloud (available online at www.ezbiocloud.net, accessed on 16 December 2025) to identify the bacterial species. Sequences showing high similarity were selected from the EZBioCloud database, and a phylogenetic tree was constructed using the Neighbor-Joining method in MEGA 11 [19]. Four isolates were identified as *Brevibacterium epidermidis* (P6), *Acinetobacter soli* (A7), *Pseudomonas parafulva* (A8), and *Pseudomonas hunanensis* (A12).

### 2.2. P. ostreatus and Selected Bacteria Interactions

Fungal culture (general). Strains *P. ostreatus*, *G. lucidum*, and *P. pulmonarius* Jinxiu were from Fuyang Mushroom Factory, Anhui Province, China. They were maintained on the plates of compound potato dextrose agar (CPDA; per liter, filtrate of 200 g boiled potato, 20 g glucose, 3 g KH_2_PO_4_, 1.5 g Mg_S_O_4_-7H_2_O, 0.05 g vitamin B, and 15 g agar [20,21]). Five plugs (5 mm in diameter) of *P. ostreatus* actively growing on the PDA plates at 25 °C were inoculated into 100 mL liquid PDA medium and grown at 25 °C for 4 d with shaking at 120 rpm. The mycelia were then homogenized twice at 3000 rpm for 5 s and used as the spawn to inoculate into the sterile substrate material (5%, *v*/*w*) and cultured in the dark at 25 °C and 80% humidity.

Co-culture on plates (screening and growth promotion). Ten isolated bacterial strains were co-cultured with *P. ostreatus* mycelium on PDA plates. A volume of 10 µL of bacterial culture in the logarithmic growth phase (OD_600_ = 0.6~0.8) was pipetted and uniformly streaked on one side of the PDA plate, 1 cm from the edge, followed by incubation at 37 °C for 2 d. Subsequently, the same size of pre-activated *P. ostreatus* hyphae was inoculated diagonally opposite to the bacterial streak, and the plates were further incubated at 28 °C for an additional 7 d. As a control for the co-culture experiment, the PDA plate was inoculated exclusively with *P. ostreatus* mycelium. All control and co-culture plates were tested in triplicate. Photographs were taken after 7 d of co-culture, and the radius of the *P. ostreatus* mycelial mass was measured using ImageJ software (version 1.53t). The relative growth promotion rate (RG) of mycelia co-cultured with bacteria, compared to those grown without bacteria, was calculated according to the following equation, where R_sample_ is the colony radius measured on the side facing the bacterial streak, and R_control_ is the colony radius of the blank control colony inoculated only with the fungal mycelial plug without bacterial treatment.

(1) $$RG(\%)=\frac{R_{sample}-R_{control}}{R_{control}}\times 100$$

#### 2.2.1. Bacterial Exopolysaccharide (EPS)

The bacterial culture suspension was harvested at an OD_600_ of 2.0, incubated in a water bath at 100 °C for 15 min, and then centrifuged at 4 °C and 8000 rpm for 15 min to collect the supernatant. A 12% trichloroacetic acid solution, prepared in a volume equal to half that of the supernatant, was added and stirred for 30 min to precipitate proteins. Following centrifugation, the supernatant was mixed with 95% ethanol at twice its volume and left overnight for precipitation. The resulting precipitate, containing bacterial EPS, was collected by centrifugation at 4 °C and 8000 rpm for 15 min [22]. The EPS mass was determined. Based on the quantity of the EPS, the precipitate was then diluted with sterile ddH_2_O to prepare solutions at concentrations of 2% (*w*/*v*), 5% (*w*/*v*), and 10% (*w*/*v*), respectively. These solutions were filtered, and 100 µL of each concentration was coated onto PDA plates. Sterile ddH_2_O served as the control. Pre-activated *P. ostreatus* hyphae of uniform size were inoculated onto the PDA plates and incubated at 28 °C. The daily growth of mycelia was recorded, and the RG was calculated.

#### 2.2.2. Bacterial Crude Proteins

The bacterial culture suspension was harvested at an OD_600_ of 2.0 and centrifuged at 8000 rpm for 20 min. The supernatant was then treated with ammonium sulfate to achieve 80% saturation and incubated at 4 °C overnight. Following centrifugation at 10,000 rpm for 20 min at 4 °C, the precipitate was resuspended in PBS buffer (0.02 M, pH 7.2), transferred into a dialysis bag, and dialyzed against 2 L of PBS buffer for desalting [23]. The dialysate was replaced after 3, 6, and 12 h of dialysis until no precipitate was observed upon titration with a 1% BaCl_2_ solution. The solution within the dialysis bag was subsequently vacuum-dried at 45 °C, and the resulting dried material was dissolved in PBS buffer to prepare crude protein solutions at mass-to-volume concentrations of 2%, 5%, and 10%, respectively. These solutions were then sterilized using a Biosharp 0.22 μm filter membrane and uniformly coated onto PDA plates. The same size of pre-activated *P. ostreatus* hyphae was inoculated onto the PDA plates and incubated at 28 °C. The diameters of the mycelia were recorded daily to calculate the RG of the mycelia.

#### 2.2.3. Test Tube Experiments

The culture substrate consisted of corn cob (72%), wheat bran (25%), and CaCO_3_ (1%). Approximately 2% lime was added to adjust the pH to 6.5, and phosphate buffer was used to adjust the moisture content to 65%. After thorough mixing, 20 g of substrate was dispensed into each test tube (30 × 150 mm), sterilized at 121 °C for 40 min, and cooled before use. For bacterial treatment, a single colony of each of the four strains was inoculated into 5 mL of LB medium and cultured overnight at 37 °C and 200 rpm until the OD_600_ reached 1.2. Then, 2% (*v*/*v*) of each culture was transferred into 100 mL of LB medium and incubated under the same conditions until the OD_600_ again reached 1.2. Each culture was divided into two portions. One portion was used directly, whereas the other was centrifuged at 8000 rpm for 15 min, and the supernatant was collected and heated in boiling water for 30 min. These bacterial preparations were added to the cooled substrate at 3%, 6%, and 10% (*v*/*w*), respectively, while LB medium added at the same ratios served as the control. A 7-day-old *P. ostreatus* mycelial plug was inoculated at the opening end of each test tube. All test tubes were incubated at 28 °C and 65% relative humidity, with four replicates per treatment. The linear mycelial growth length was measured after 7 d.

#### 2.2.4. Cultivation in Bags

The culture substrate was composed of corn cob (72%), wheat bran (25%), and CaCO_3_ (1%). Lime (2%) was added to adjust the substrate pH to 6.5. Approximately 600 g of sterilized substrate was placed into polyethylene bags measuring 16 × 18 cm. A bacterial culture suspension was prepared, adjusted to a final concentration of 10^8^ CFU/mL, and incorporated into the substrate at a ratio of 10% (*v*/*w*), followed by thorough mixing. LB medium served as the control. Five actively growing plugs (5 mm in diameter) of *P. ostreatus*, obtained from a PDA plate incubated at 25 °C, were transferred into 100 mL of liquid PDA medium and cultured at 25 °C for 4 d with shaking at 120 rpm. The resulting mycelia were homogenized twice at 3000 rpm for 5 s and added to the substrate bags at 5% (*v*/*w*) as the fungal inoculum. Subsequently, the inoculated substrate bags were cultured in a controlled environment at 28 °C and 65% relative humidity, under a 12 h light/12 h dark cycle to assess mycelial growth and final mushroom yield [20].

##### Incubation Time (Mycelial Colonization Time)

The time required for complete mycelial colonization of the substrate bag was recorded (in days). Full colonization was determined visually when the mycelium had permeated the entire bag.

##### Basidiomata Production

After full colonization, bags were maintained under the same conditions for basidioma. Parameters recorded included number of basidiomata per bag, fresh weight of basidiomata per bag (final yield), and basidiomata size.

##### Enzymatic Activities

The relative lignocellulosic enzymatic activities of *P. ostreatus* during mycelial growth in substrate were estimated to evaluate the fungal performance at 15 d of culture. Twenty grams of each sample were randomly collected and transferred to flasks containing 100 mL of deionized water, then blended at 4 °C in the dark overnight. The resulting mixtures were filtered to remove residues and then centrifuged at 8000× *g* for 10 min to obtain the supernatant for enzymatic activity analysis. Laccase (Lac) activity was determined using 2,2′-azino-bis (3-ethylbenzothiazoline-6-sulfonate) (ABTS) (0.5 mM) as the substrate, following the method of Bourbonnais and Paice [24]. Lignin peroxidase (LiP) activity was measured based on the oxidation of veratryl alcohol to veratraldehyde, as described by Arora [25]. Manganese peroxidase (MnP) was quantified in the presence of MnSO_4_ (0.5 mM), 2,6-dimethylphenol (2,6-DMP) (1.0 mM), and H_2_O_2_, according to the protocol of Wariishi et al. [26].

### 2.3. Bacteria Volatile Organic Compounds (VOCs)

#### 2.3.1. Preliminary Experiments (Bi-Plate Co-Culture for VOC Effects)

Bacteria and fungi were co-cultivated on bi-plates (9 cm in diameter) containing PDA and LB media. Two parallel bacterial inoculation lines were established at distances of 1.5 cm and 3 cm from the edge of each dish. On the fungal side, mycelial plugs (0.5 cm in diameter) from a pre-cultured *P. ostreatus* were positioned 2.5 cm from the barrier [10]. Bacterial strains were inoculated two days prior to fungal inoculation to allow for full development. Bi-plates inoculated with fungi only were used as a control. All plates were sealed with parafilm and incubated in the dark at 28 °C. Each treatment, including the control, was replicated at least three times. Mycelial radial growth and the RG were determined as described above.

#### 2.3.2. Pseudomonas Hunanensis Strain A12 VOC

##### Identification of VOCs via GC-MS

VOCs produced by bacteria and *P. ostreatus* were analyzed using a bi-plate system. It was clarified that a single bacterial streak was used, and a plastic barrier separated the bacterial side from the fungal side. In the control, sterile medium replaced the bacterial streak. Following the drilling of small openings on one side of the Petri dish using a needle, VOCs were collected via solid-phase microextraction (SPME) with divinylbenzene-carboxen-polydimethylsiloxane (50/30 µm DVB/CAR/PDMS) fiber tips. From day 4 post-inoculation, VOCs were sampled directly from the headspace above the culture in the crystallization dish at 25 °C for a duration of 24 h. For GC-MS analysis, a 7890 A gas chromatograph (Agilent Technologies, Waldbronn, Germany) equipped with an Agilent VF-WAXms column (30 m × 0.25 mm, 0.25 µm) was coupled to an Agilent 5975 C MSD (Agilent Technologies, Santa Clara, CA, USA). Helium was used as the carrier gas at a constant flow rate of 1.2 mL/min. Mass spectra were obtained in the range of 33–300 *m*/*z*. Electron impact ionization (70 eV) was performed with an ion source temperature of 230 °C. SPME fibers were introduced into the GC injector for thermal desorption in splitless mode for 1 min, with the injector temperature maintained at 250 °C. The GC oven temperature program was set as follows: initial temperature of 40 °C (held for 3 min), ramped at 5 °C/min to 240 °C (held for 7 min). The VOCs were identified by comparing the obtained mass spectra with reference spectra in the NIST14 database.

##### Assay of Pure VOCs on *P. ostreatus* Growth

Experiments were performed using a bi-plate system as described above. Instead of adding bacterial cultures into one compartment, varying concentrations of pure chemical substances were dissolved in methanol and applied to one side of the barrier, while *P. ostreatus* was cultivated on the opposite side. A bi-plate containing only an equivalent volume of methanol and inoculated with *P. ostreatus* mycelium was used as the control. All plates were sealed with parafilm and incubated at 28 °C in the dark. Experiments were performed at least in triplicate. Mycelial radial growth was measured daily, and the relative growth rate was calculated as described above.

### 2.4. Selected Bacteria Interactions with G. lucidum and P. pulmonarius

The co-culture of the four bacteria (P6, A7, A8, A12), which exhibited positive effects with *G. lucidum* and *P. pulmonarius*, was conducted in the same manner as described for *P. ostreatus* in Section 2.2. All control and co-culture plates were tested in triplicate. Photographs were taken after 7 d of co-culture, and the radius of the mycelial mass was measured using ImageJ software. RG was calculated according to the same equation.

### 2.5. Statistical Analyses

Statistical analyses and figure preparation were performed using Excel 2019, IBM SPSS Statistics (version 27), and GraphPad Prism (version 9.0). For comparisons between two groups, Student’s *t*-test was used. For comparisons among three or more groups, one-way analysis of variance (ANOVA) was performed, followed by Dunnett’s multiple comparison test when each treatment group was compared with the corresponding control group. Statistical significance was defined as *p* < 0.05. In the figures, significance levels are indicated as follows: * *p* < 0.05, ** *p* < 0.01, *** *p* < 0.001, **** *p* < 0.0001; ns, not significant.

## 3. Results

### 3.1. Screening Bacterial Strains from P. ostreatus-Cultivated Bags for Mushroom Growth-Promotion

**Figure 1:**
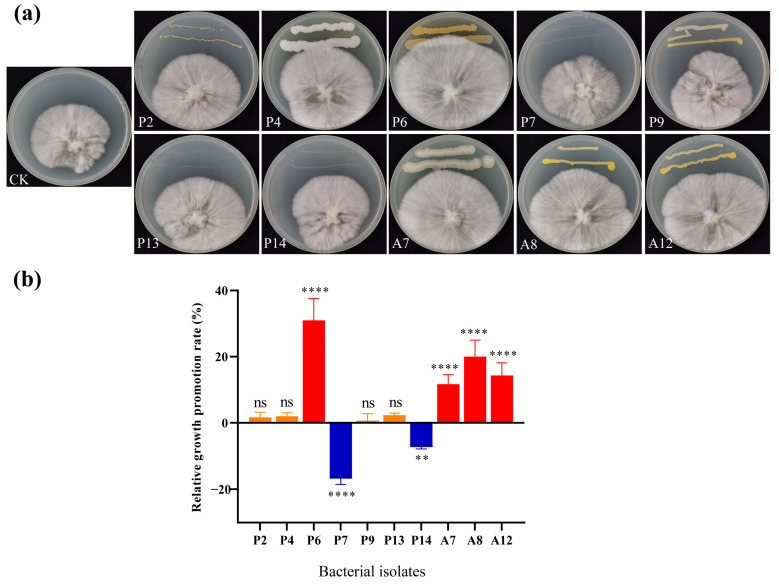
Co-culture interactions between *P. ostreatus* and bacterial isolates recovered from the cultivation bags of commercial *P. ostreatus* production. (**a**) Interactions on potato dextrose agar (PDA). CK represents the control. Bacterial isolates P2, P4, *Brevibacterium epidermidis* P6, P7, P9, P13, P14, *Acinetobacter soli* A7, *Pseudomonas parafulva* A8, and *Pseudomonas hunanensis* A12 were streaked on the upper side of the plate, and a *P. ostreatus* mycelial plug was placed on the lower side. Images were taken 7 days after fungal inoculation. (**b**) The RG of *P. ostreatus* co-cultivated with bacterial strains as compared with the control. Data are presented as the mean ± standard deviation, *n* = 3. Statistical significance was determined using the original mycelial radius data via one-way ANOVA followed by Dunnett’s multiple comparison test against the CK group. ** *p* < 0.01; **** *p* < 0.0001; ns, not significant.

A total of 24 bacterial isolates were obtained from *P. ostreatus* substrate samples collected at the half-colonization and full-colonization stages, and the relevant information has been provided in Supplementary Figure S1. After excluding isolates with obvious pathogenic potential based on colony morphology and hemolytic activity, ten bacterial isolates were selected for co-culture experiments with *P. ostreatus*. As shown in Figure 1, four bacterial strains markedly enhanced the mycelial growth of *P. ostreatus*. Specifically, strain P6 demonstrated the highest growth promotion rate at 31%, followed by A8 at 20%, A12 at 14.3%, and A7 at 11.67%. In contrast, four strains (P2, P4, P9, and P13) exhibited no significant influence on mycelial growth, while two strains (P7 and P14) displayed inhibitory effects. The four growth-promoting bacterial strains were identified as *B. epidermidis* P6 (P6), *A. soli* A7 (A7), *P. parafulva* A8 (A8), and *P. hunanensis* A12 (A12) based on 16S rRNA gene sequence similarities (Figure S2). The non-promoting or inhibitory strains were not further characterized, as they were not the focus of this study.

### 3.2. Four Isolated Bacterial Strains Shorten the Mycelial Colonization Time in Substrates and Increase Fresh Mushroom Production

**Figure 2:**
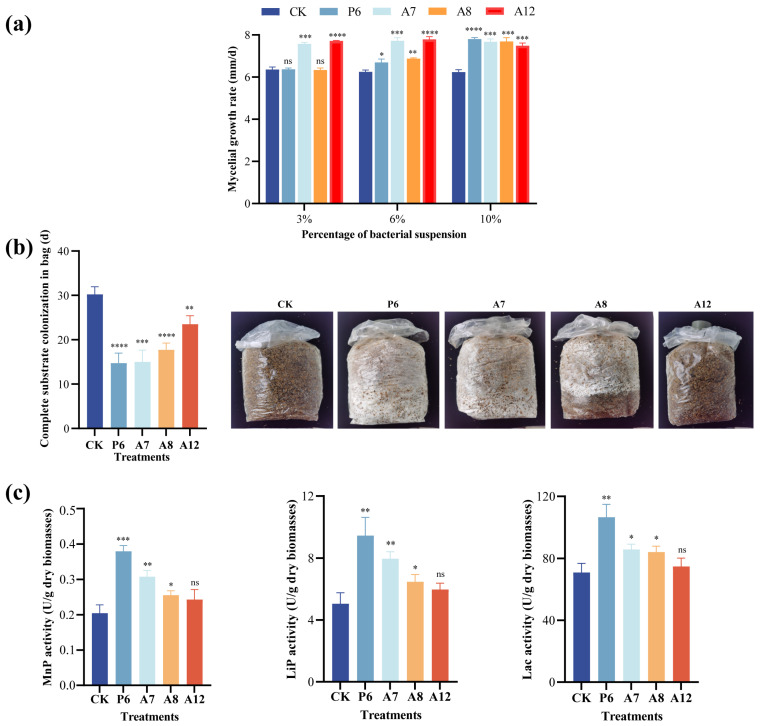
Four isolated bacterial strains shorten the mycelial colonization time of *P. ostreatus* in substrates. (**a**) The mycelial growth rates of *P. ostreatus* treated with different bacterial culture suspension in test tube experiments. (**b**) Complete mycelial colonization time and representative cultivation-bag images under different treatments. (**c**) The ligninolytic enzyme activities of *P. ostreatus* mycelia at 15 d of incubation. Statistical significance was determined via one-way ANOVA followed by Dunnett’s multiple comparison test against the corresponding CK group. * *p* < 0.05; ** *p* < 0.01; *** *p* < 0.001; **** *p* < 0.0001; ns, not significant.

In test tube experiments with a 3% addition of the growth-promoting strains’ bacterial culture suspension, the mycelial colonization time of *P. ostreatus* was significantly reduced and the radial growth was enhanced (Figure 2a), the inclusion of bacterial solutions from strains A7 and A12 significantly increased the mycelial growth rate of *P. ostreatus*. Compared to the control group (CK), which exhibited an average daily growth rate of 6.35 mm/d, the addition of strain A7 resulted in a growth rate of 7.57 mm/d, while strain A12 resulted in a growth rate of 7.71 mm/d. In contrast, the addition of strain P6 or A8 did not significantly enhance the mycelial growth rate. When the addition ratio of the bacterial culture suspension was increased to 10% (Figure 2a), all four bacterial strains significantly enhanced the mycelial growth of *P. ostreatus* mycelium, with daily growth rates of 7.80, 7.68, 7.69, and 7.49 mm/d observed for the addition of strains P6, A7, A8, and A12, respectively, compared to 6.27 mm/d in the CK.

The addition of the fermentation broth of the four growth-promoting strains at a concentration of 10% (*v*/*w*) significantly reduced the mycelial colonization time and enhanced the mycelial growth rate within bags. After 15 d of incubation, observations of mycelial development revealed that the CK group had not yet exhibited obvious mycelial extension in bags, whereas the P6 and A7 bacterial treatments had achieved full mycelial colonization. Strain A8 treatment led to approximately two-thirds of mycelial colonization, while strain A12 treatment showed performance similar to the CK group, with no observable downward mycelial growth (Figure 2b). In terms of the time required for full mycelial colonization in the substrate, the CK group took 30.2 ± 1.8 days. In contrast, the treatment of strains P6 and A7 demonstrated the most significant growth-promoting effects. Specifically, strain P6 treatment required 14.0 ± 4 days, strain A7 treatment required 15.0 ± 3.0 days, strain A8 treatment required 17.8 ± 2.2 days, and strain A12 treatment required 23.5 ± 1.5 days to colonize the substrate fully (Figure 2b).

As shown in Figure 2c, strains P6, A7, and A8 treatment groups showed significantly higher LiP, MnP, and Lac activities than the CK group. We measured these ligninolytic enzyme activities after 15 days of incubation to evaluate mycelial growth performance. *P. ostreatus* is a widely recognized lignocellulose-degrading fungus that possesses a robust enzymatic system including LiP, MnP, and Lac, which helps it grow on various substrates [27]. The increased enzyme activities were observed in treatments that also showed faster substrate colonization.

**Figure 3:**
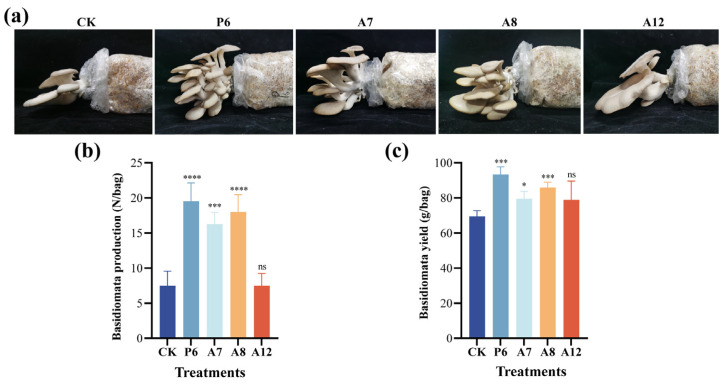
Four growth-promoting bacterial strains enhance basidioma production in *P. ostreatus*. (**a**) Growth phenotype of *P. ostreatus* co-cultivated with different bacterial strains in cultivation bags. (**b**) The number of *P. ostreatus* basidiomata per bag. (**c**) Final yield of fresh basidiomata per bag. The four growth-promoting bacterial strains were *B. epidermidis* strain P6, *A. soli* strain A7, *P. parafulva* strain A8, and *P. hunanensis* strain A12. Data are presented as the mean ± standard deviation, *n* = 3. Statistical significance was determined via one-way ANOVA followed by Dunnett’s multiple comparison test against the CK group. * *p* < 0.05; *** *p* < 0.001; **** *p* < 0.0001; ns, not significant.

The addition of growth-promoting bacterial strains also positively influenced the development of *P. ostreatus* basidiomata (Figure 3). Compared with the CK group, the application of strains P6, A7, and A8 significantly increased the number of basidiomata. In contrast, strain A12 did not affect basidioma number but resulted in larger individual size (Figure 3a,b). After 7 d from the initiation of spawn running, the final yield of *P. ostreatus* basidiomata was 69.46 ± 3.94 g in the CK group, compared to 93.31 ± 4.76 g in the P6 group, 79.40 ± 4.7 g in the A7 group, 85.90 ± 3.3 g in the A8 group, and 78.94 ± 8.5 g in the A12 group (Figure 3c).

### 3.3. Effects of Crude Proteins and EPSs from Bacterial Strains on the Mycelial Growth of P. ostreatus

**Figure 4:**
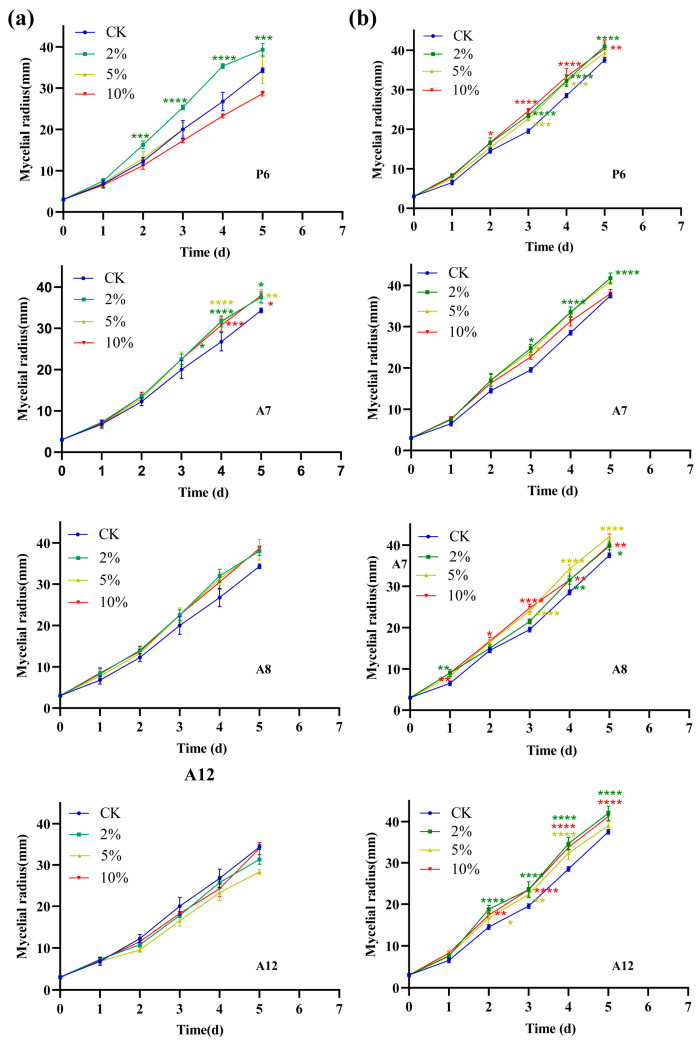
Effects of extracellular crude protein extracts and exopolysaccharides (EPSs) from four selected bacterial isolates on the mycelial growth of *P. ostreatus*. The effects of extracellular crude proteins (**a**) and EPSs (**b**) produced by four growth-promoting bacterial isolates on the mycelial growth of *P. ostreatus* were evaluated using Petri dish assays. CK represents the PDA control without crude protein or EPS treatment. P6, A7, A8, and A12 represent the selected bacterial isolates, namely *B. epidermidis* P6, *A. soli* A7, *P. parafulva* A8, and *P. hunanensis* A12, respectively. The concentrations of 2%, 5%, and 10% indicate the final concentrations of crude protein extracts or EPS solutions used in the assays. Mycelial growth was evaluated by measuring the mycelial radius. Data are presented as the mean ± standard deviation, *n* = 3. Statistical significance was determined at each time point via one-way ANOVA followed by Dunnett’s multiple comparison test against the CK group. * *p* < 0.05; ** *p* < 0.01; *** *p* < 0.001; **** *p* < 0.0001.

Bacteria can secrete soluble compounds or VOCs capable of influencing fungal growth. The effects of extracellular crude proteins and EPS produced by bacterial strains on the mycelial growth of *P. ostreatus* were initially evaluated using Petri dishes. The results indicated that a 2% concentration of crude protein from strain P6 could significantly enhance the mycelial growth rate of *P. ostreatus*. In contrast, a 5% concentration not only failed to promote growth but also exerted inhibitory effects on *P. ostreatus* mycelium growth (Figure 4a). Crude protein solutions from strains A7 and A8 promoted mycelial growth across all tested concentrations, with no significant differences observed among concentration levels. Conversely, crude protein extracted from strain A12 did not enhance mycelial growth at any concentration (Figure 4a).

After extracting EPSs from the four growth-promoting bacterial strains, the EPS solutions were diluted with sterile water to concentrations of 2%, 5% and 10%, respectively. These solutions were applied to assess their effects on the mycelial growth of *P. ostreatus*. The addition of EPS from all strains resulted in significantly greater mycelial growth compared to the CK group, with no obvious difference among concentrations (Figure 4b).

### 3.4. Effects of VOCs from Bacterial Strains on the Mycelial Growth of P. ostreatus

**Figure 5:**
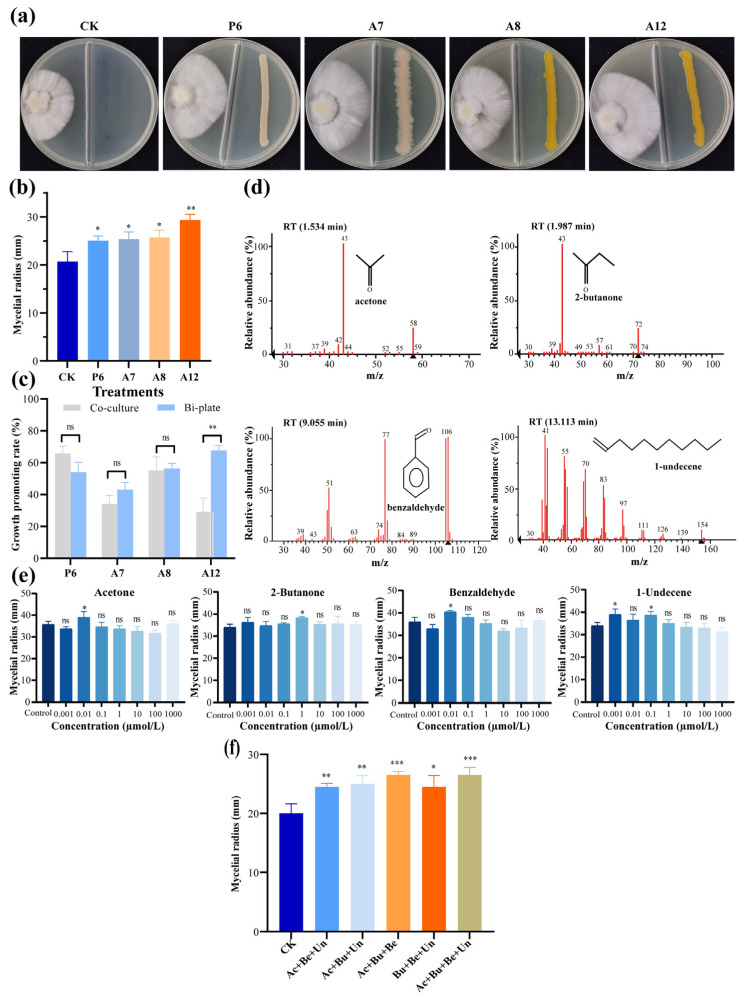
Interactions between *P. ostreatus* and volatile organic compounds (VOCs) produced by four selected bacterial strains. The VOCs were produced by *B. epidermidis* strain P6, *A. soli* strain A7, *P. parafulva* strain A8, and *P. hunanensis* strain A12. (**a**) Effects of 4-day exposure to bacterial VOCs on the mycelial growth of *P. ostreatus* in a bi-plate assay. (**b**) Mycelial radius of *P. ostreatus* after 4 days of exposure to bacterial VOCs in the bi-plate assay. (**c**) Comparison of the RG between the bi-plate assay and the single-plate co-culture system. (**d**) GC-MS analysis of VOCs produced by strain A12 in the bi-plate co-culture system with *P. ostreatus*. (**e**) Mycelial growth of *P. ostreatus* after 7 days of exposure to different concentrations of acetone, 2-butanone, benzaldehyde, and 1-undecene, with methanol used as the solvent control. (**f**) Synergistic effects of the four pure VOCs on the mycelial growth of *P. ostreatus*. Data are presented as the mean ± standard deviation, *n* = 3. Statistical significance was determined via one-way ANOVA followed by Dunnett’s multiple comparison test against the corresponding control, except for panel (**c**), where Student’s *t*-test was used to compare the bi-plate assay with the single-plate co-culture system for each bacterial strain. * *p* < 0.05; ** *p* < 0.01; *** *p* < 0.001; ns, not significant.

The effects of VOCs produced by bacterial strains on the mycelial growth of *P. ostreatus* were subsequently evaluated using bi-plate assays. After 4 d of incubation, the radial growth of the control CK mycelium was 20.7 ± 2.1 mm, whereas the mycelial radii in the presence of VOCs from strains P6, A7, A8, and A12 reached 25.0 ± 1.0 mm, 25.3 ± 1.5 mm, 25.7 ± 1.5 mm, and 29.3 ± 1.2 mm, respectively (Figure 5a and Figure 5b). Compared with the mycelial growth promotion observed during single-plate co-culture, the VOCs emitted by strains P6, A7, and A8 exhibited comparable enhancing effects. In contrast, the VOCs produced by strain A12 significantly increased the mycelial growth rate of *P. ostreatus* mycelium by approximately 39% relative to the single-plate co-culture system (Figure 5c). Furthermore, consistent with the increased loading of strain A12, the mycelial growth rate of *P. ostreatus* was enhanced, reaching a maximum at 50 µL of bacterial solution and declining at 70 µL. Overall, the gaseous phase generated by strain A12 contains certain substances that effectively promote the mycelial growth of *P. ostreatus* at appropriate concentrations.

### 3.5. Characterization of VOCs from Strain A12 and Their Effects on the Mycelial Growth of P. ostreatus

The VOCs emitted during the single-culture of strain A12, the single-culture of *P. ostreatus*, and the bi-plate co-culture of A12 and *P. ostreatus* were collected and analyzed using GC-MS. Compared to the VOC profiles of the individual single cultures, the signal intensities of acetone and 2-butanone were significantly enhanced in the co-culture system. Additionally, benzaldehyde and 1-undecene were detected in both the single culture of strain A12 and the bi-plate co-culture of A12 and *P. ostreatus* (Figure 5d). These four compounds were individually diluted to various concentrations to evaluate their effects on the mycelial growth of *P. ostreatus*. Optimal growth promotion was observed at a concentration of 0.01 µmol/L for acetone and benzaldehyde, 1 µmol/L for 2-butanone, and 0.001 µmol/L for 1-undecene (Figure 5e). Furthermore, the synergistic effects of these VOCs were evaluated by combining them in groups of three at their respective optimal concentrations. All combinations exhibited the most pronounced growth-promoting effects during the early growth stage. Notably, the combination of acetone, 2-butanone, and benzaldehyde yielded the highest stimulation effect of mycelial growth, achieving an approximate promotion rate of about 33%, which was comparable to the impact of the four-component mixture (Figure 5f).

### 3.6. The Bacterial Strains Exhibit a Growth-Promoting Effect on the Mycelial Growth of G. lucidum and P. pulmonarius

**Figure 6:**
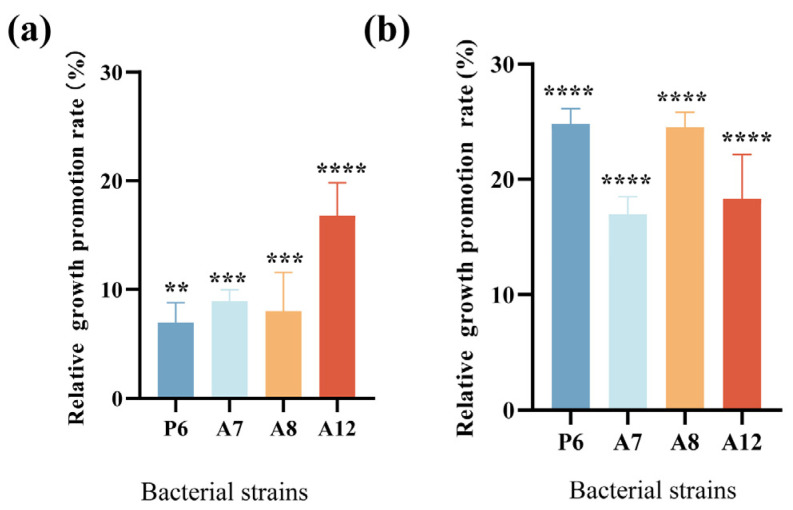
RG in co-cultures of *G. lucidum* and *P. pulmonarius* with four selected bacterial strains. Relative growth promotion rate of *G. lucidum* (**a**) and *P. pulmonarius* (**b**) co-cultured with *B. epidermidis* strain P6, *A. soli* strain A7, *P. parafulva* strain A8, and *P. hunanensis* strain A12. Data are presented as the mean ± standard deviation, *n* = 3. Statistical significance was determined using the original mycelial radius data via one-way ANOVA followed by Dunnett’s multiple comparison test against the CK group. ** *p* < 0.01; *** *p* < 0.001; **** *p* < 0.0001.

To further expand the range of mushroom species capable of interacting with these four bacterial strains, *G. lucidum* and *P. pulmonarius* were employed to evaluate their mycelial growth rates in the presence and absence of bacterial strains. As shown in Figure 6, the mycelial growth of both fungal species was accelerated. Specifically, for *G. lucidum*, strain A12 exhibited the highest growth promotion rate of 16.82%, while the other strains showed promotion rates ranging from approximately 7% to 9%. For *P. pulmonarius*, strains P6 and A8 achieved growth promotion rates of nearly 24%, whereas strains A7 and A12 demonstrated rates of approximately 17–18%.

## 4. Discussion

*P. ostreatus* is a commercially important edible mushroom, the growth and development of which are closely associated with the microbial composition of its cultivation bags [28,29]. This study identified four bacterial strains isolated from *P. ostreatus* cultivation bags that significantly enhance mycelial growth, accelerate substrate colonization, elevate lignin-degrading enzyme activities, and increase basidiomata yield. Notably, *B. epidermidis* P6 and *P. parafulva* A8 exhibited the most pronounced promoting effects. These beneficial effects are primarily mediated through the synergistic action of soluble metabolites and VOCs. Moreover, the growth-promoting activity of these strains extends to other fungal species, including *G. lucidum* and *P. pulmonarius*, indicating their potential for broad application in commercial mushroom cultivation.

Among the four bacterial strains, two belong to the genus *Pseudomonas*. These findings are consistent with numerous previous studies showing that members of the genus *Pseudomonas* can significantly promote fungal mycelial expansion, primordium initiation, and basidioma maturation through metabolic interactions and cross-kingdom signaling, as well as modulate post-harvest microbial dynamics and extend shelf life [30,31,32,33]. Furthermore, the growth-promoting capacity of *Pseudomonas* is not restricted to fungi, as it is also well-documented in plants [33]. However, not all *Pseudomonas* species exert beneficial effects; certain strains, such as *P. gingeri* and *P.* B2, have been reported to be pathogenic to *Agaricus bisporus* [34,35]. Importantly, this study highlights that even within the same genus, different species may employ distinct mechanisms and elicit divergent phenotypic responses in fungi, since *P. parafulva* A8 markedly increased the number of *P. ostreatus* basidiomata, whereas *P. hunanensis* A12 primarily influenced its basidioma size.

This study also demonstrates that *B. epidermidis* P6 and *A. soli* A7 exert significant growth-promoting effects on *P. ostreatus*, *G. lucidum*, and *P. pulmonarius*. For *Brevibacterium*, direct evidence for growth promotion in cultivated mushroom systems remains limited. However, members of this genus have been reported in spent mushroom compost, termite fungus-comb systems associated with basidiome formation, and bacterial communities associated with mushroom developmental stages, suggesting a potential ecological role in mushroom-associated microbial systems [36]. *B. frigoritolerans* has been isolated from spent mushroom compost and exhibits lead resistance [37]. In addition, *Brevibacterium* species have been found to be abundant in *Termitomyces* basidiomes [38]. Direct evidence for the growth-promoting effect of *Acinetobacter* in edible mushroom systems is still limited. However, *Acinetobacter*-associated strains have been reported to stimulate mycelial growth in *Polyporus umbellatus*, and members of this genus have also been detected in A. bisporus casing soil and *P. ostreatus* substrate preparation systems. In addition, Wei et al. reported that *Acinetobacter* became the predominant genus at the end stage of composting cultivation of *Pleurotus floridanus*, suggesting that this genus may participate in substrate transformation and play beneficial roles in mushroom-associated microbial systems [39,40,41]. *Acinetobacter* species appear to play an important role in substrate degradation during the cultivation of *P. ostreatus* and have also been detected in association with *Flammulina filiformis* and its production environment [42,43]. Nevertheless, despite increasing interest in microbial inoculants for agriculture and mycoculture, current research largely focuses on complex microbial consortia, leaving the specific mechanisms underlying strain-specific growth promotion insufficiently elucidated.

Results from petri-dish, tube-based, and substrate bag experiments indicate that the growth-promoting effects of the isolated bacterial strains are both strain-specific and concentration-dependent. A wide range of microorganisms can engage in diverse interactions, spanning from antagonistic to mutually beneficial relationships between bacteria and fungi [1]. Strains P7 and P14 exhibited inhibitory effects on the mycelial growth of *P. ostreatus*, suggesting a potential antagonistic interaction with this fungus. At an inoculum concentration of 3%, only strains A7 and A12 significantly enhanced mycelial growth rates. In contrast, at a 10% inoculation level, all four strains exhibited significant activity, indicating that a threshold population density may be required for observable effects. In substrate bag experiments, strains P6, A7, and A8 substantially reduced the time required for complete mycelial colonization of the substrate, thereby increasing the yield of mushrooms. It has been reported that the increase in mushroom yield induced by bacteria may be due to their synergistic effect on mycelial growth stimulation [44]. For example, *Bacillus subtilis* significantly boosts mushroom yield when lower concentrations of bacterial broth are applied [45]. Inoculation of *Pseudomonas* sp. P7014 not only increases basidioma production, but also leads to an earlier initiation of *P. eryngii* primordia [46]. From a practical perspective, shortening the colonization phase may reduce the overall cultivation duration and could potentially minimize the risk of contamination in commercial production settings, although this possibility was not directly tested in the present study.

These growth-promoting microorganisms can also facilitate the degradation of substrates and release various monosaccharides, which serve as readily available carbon sources for mushrooms, thereby accelerating mycelial growth and increasing yield [47]. In this study, EPS and crude protein extracts from growth-promoting bacteria were demonstrated to enhance the mycelial growth of *P. ostreatus* mycelia. These bacterial extracts may also reduce the risk of contamination during cultivation, as numerous studies have reported that the bacterial crude proteins exhibit antagonistic activity against pathogenic bacteria and fungi [23,48,49]. Furthermore, VOCs produced by all four bacterial strains, particularly strain A12, were capable of diffusing through the air in a bi-plate system, acting in synergy with EPS and crude protein to promote *P. ostreatus* mycelial development. VOCs play an important role in fungal-bacterial interactions, where they can either positively or negatively influence each other’s growth dynamics [50,51]. While most studies indicate that bacterial VOCs inhibit fungal growth or development, reports on VOCs with growth-promoting properties are rare [4,10,15]. Notably, VOCs from *Pseudomonas* species are predominantly associated with antifungal or antibacterial activities against human or plant pathogens [52,53]. In contrast, the VOCs emitted by *P. parafulva* A8 and *P. hunanensis* A12 showed a growth-stimulatory effect on *P. ostreatus* here.

Few studies have identified bacterial VOCs that promote fungal growth. Briard et al. demonstrated that VOCs emitted by *P. aeruginosa* significantly increased the biomass of *A. fumigatus*, with dimethylsulphide potentially serving as the active stimulatory compound [15]. *Bacillus* sp. M48F has been shown to release a substantial concentration of 2,5-diisopropylpyrazine, which promotes mushroom mycelial growth [10,15]. However, when the compound was tested for its effects on the development of *P. ostreatus*, the results indicated that it alone is not responsible for the full growth-promoting effect observed with *Bacillus* sp. M48F, suggesting the involvement of additional compounds [10]. Similarly, in this study, the effects of acetone and 2-butanone—two pure substances detected in bacterial–fungal co-cultures—on *P. ostreatus* mycelia were evaluated and found to be significantly less effective than those induced by strain A12. This suggested that these two compounds are insufficient to account for significant mycelial growth promotion, and that other growth-stimulating volatiles may be specifically produced by strain A12. Therefore, benzaldehyde and 1-undecene were individually and combinatorially tested for their effects on *P. ostreatus* growth. The combined application of these four substances resulted in a significantly enhanced growth-promoting effect compared to individual treatments, although it remained lower than the effect elicited by live A12. These findings imply that synergistic interactions among multiple VOCs and soluble substances contribute to the mechanism underlying bacterial–fungal interactions in this system.

## 5. Conclusions

This study isolated and characterized four native bacterial strains from the cultivation bags of *P. ostreatus* that significantly promoted mycelial growth and mushroom yield. The main findings are as follows:

Strain-specific promotion: *B. epidermidis* P6, *A. soli* A7 and *P. parafulva* A8 shortened substrate colonization time from approximately 30 days to 14–18 days and increased basidioma yield, whereas *P. hunanensis* A12 primarily enhanced individual basidioma size rather than number.

Multiple mechanisms: Growth promotion was mediated by extracellular polysaccharides (all four strains), crude proteins (P6, A7, A8), and volatile organic compounds (all four strains, especially A12). The VOCs acetone, 2-butanone, benzaldehyde, and 1-undecene from A12 acted synergistically to enhance mycelial growth.

Broad-spectrum activity: All four strains also promoted mycelial growth of *G. lucidum* and *P. pulmonarius*, suggesting potential applicability across multiple commercial mushroom species.

Practical implications: These bacterial strains represent promising candidates for developing microbial inoculants to improve the efficiency of commercial mushroom cultivation, particularly by shortening the colonization phase and increasing yield.

Future perspectives: Validation under large-scale production conditions, formulation of stable inoculants, and molecular dissection of the growth-promoting pathways (e.g., genes involved in EPS or VOC biosynthesis) are warranted to enable precise application in mushroom farming.

## Data Availability

The DNA sequences of the 16S rRNA gene for molecular identification of the four bacterial strains presented in this study are openly available in GenBank at https://www.ncbi.nlm.nih.gov/nuccore, accessed on 20 May 2026, reference number PV366723, PX427491, PX427734, and PV366630.

## Supplementary Materials

The following supporting information can be downloaded at: https://www.mdpi.com/article/10.3390/microorganisms14061181/s1, Figure S1: Morphology of bacterial strains isolated from fungal cultivation bags; Figure S2: Identification of four selected bacterial strains based on 16S rRNA gene analysis. (a) Phylogenetic tree of the four selected bacterial strains constructed based on 16S rRNA gene sequences. The selected strains were identified as *B. epidermidis* P6, *P. parafulva* A8, *A. soli A7*, and *P. hunanensis* A12. (b) Agarose gel electrophoresis of 16S rRNA gene PCR products from strains P6, A12, A7, and A8.
