# Supplementary material for: Native Bacteria Associated with Mushroom Cultivation Promote Mushroom Growth Through Multiple Mechanisms
Source: Microorganisms. 2026 May 24;14(6):1181. doi: 10.3390/microorganisms14061181 (PMC13304464; doi:10.3390/microorganisms14061181)
Supplement: Supplementary file 1 [file microorganisms-14-01181-s001.zip › microorganisms-4264215-supplementary.pdf]

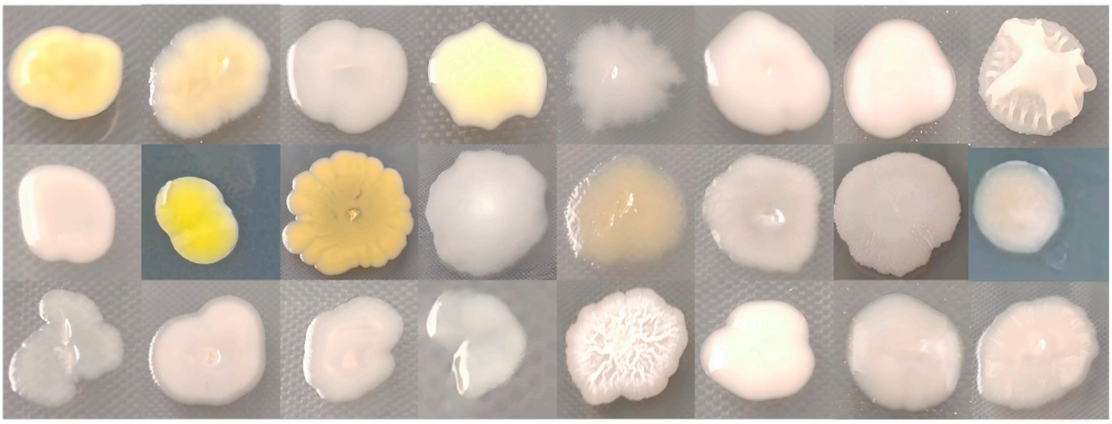

Figure S1. Morphology of bacterial strains isolated from fungal cultivation bags.

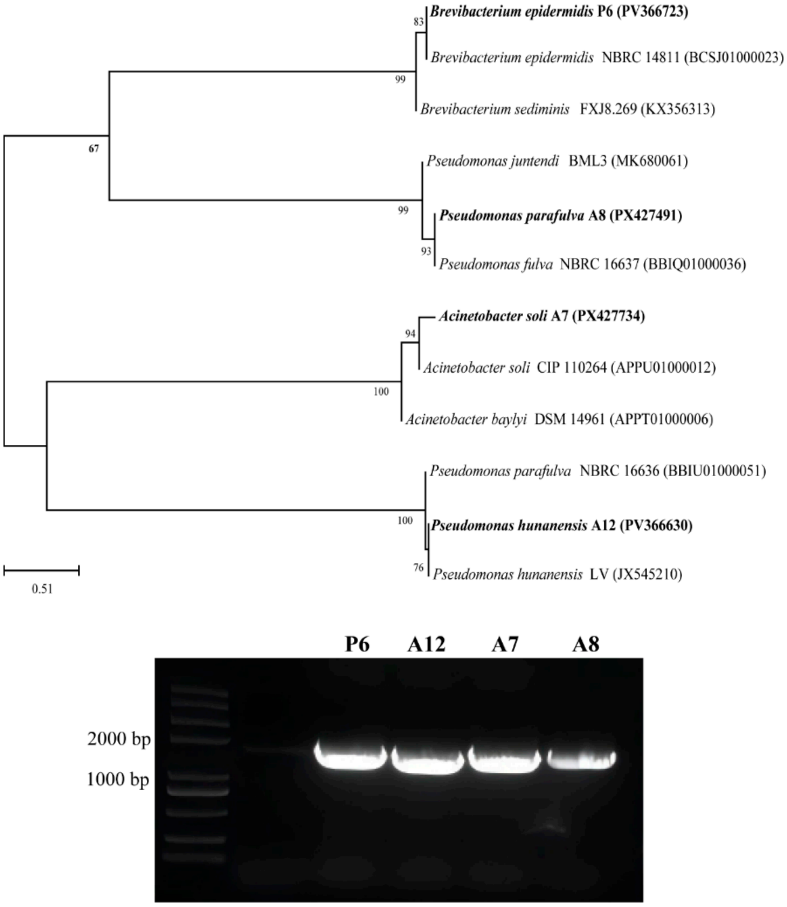

Figure S2. Identification of four selected bacterial strains based on 16S rRNA gene analysis. (a) Phylogenetic tree of the four selected bacterial strains constructed based on 16S rRNA gene sequences. The selected strains were identified as *B. epidermidis* P6, *P. parafulva* A8, *A. soli* A7, and *P. hunanensis* A12. (b) Agarose gel electrophoresis of 16S rRNA gene PCR products from strains P6, A12, A7, and A8.
